# An In-Silico Pipeline for Rapid Screening of DNA Aptamers against Mycotoxins: The Case-Study of Fumonisin B1, Aflatoxin B1 and Ochratoxin A

**DOI:** 10.3390/polym12122983

**Published:** 2020-12-14

**Authors:** Fulvio Ciriaco, Vincenzo De Leo, Lucia Catucci, Michelangelo Pascale, Antonio F. Logrieco, Maria C. DeRosa, Annalisa De Girolamo

**Affiliations:** 1 Department of Chemistry, University of Bari, Via Orabona 4, 70126 Bari, Italy; vincenzo.deleo@uniba.it (V.D.L.); lucia.catucci@uniba.it (L.C.); 2 Institute of Sciences of Food Production (ISPA), CNR-National Research Council of Italy, Via G. Amendola 122/O, 70126 Bari, Italy; michelangelo.pascale@ispa.cnr.it (M.P.); antonio.logrieco@ispa.cnr.it (A.F.L.); 3 Department of Chemistry, Carleton University, 1125 Colonel by Drive, Ottawa, ON K1S 5B6, Canada; maria_derosa@carleton.ca

**Keywords:** ssDNA aptamers, in-silico approach, binding affinity, mycotoxins, fumonisin B1, aflatoxin B1, ochratoxin A

## Abstract

Aptamers are single-stranded oligonucleotides selected by SELEX (Systematic Evolution of Ligands by EXponential Enrichment) able to discriminate target molecules with high affinity and specificity, even in the case of very closely related structures. Aptamers have been produced for several targets including small molecules like mycotoxins; however, the high affinity for their respective target molecules is a critical requirement. In the last decade, the screening through computational methods of aptamers for their affinity against specific targets has greatly increased and is becoming a commonly used procedure due to its convenience and low costs. This paper describes an in-silico approach for rapid screening of ten ssDNA aptamer sequences against fumonisin B1 (FB1, *n* = 3), aflatoxin B1 (AFB1, *n* = 2) and ochratoxin A (OTA, *n* = 5). Theoretical results were compared with those obtained by testing the same aptamers by fluorescent microscale thermophoresis and by magnetic beads assay for their binding affinity (*K_D_*) revealing a good agreement.

## 1. Introduction

Aptamers are nucleic acid biopolymers and represent a new generation of receptors that are selected in vitro by the Systematic Evolution of Ligands by EXponential Enrichment (SELEX) [1,2]. Aptamers are composed of single-stranded DNA (ssDNA) or RNA usually in the region of 35–100 nucleotides in length and have the ability to recognize different classes of targets such as mycotoxins, amino acids, antibiotics, pesticides, proteins and even whole cells with high specificity and affinity [3,4,5,6]. Compared to antibody generation, SELEX allows greater control over binding conditions and allows selection under non-physiological conditions. In addition to the potential for low production costs, benefits of aptamers with respect to antibodies include short time of development process, detection of non-immunogenic small molecules, stability during storage and improved tolerance to solvents or extreme pH or ionic strength.

The past few years have seen a dramatic increase in aptamer development and interest for diagnostic and therapeutic applications [7]. Recently, there has been an increase in demand for the detection of small molecules (<900 g/mol) like mycotoxins, secondary metabolites produced by filamentous fungi frequently contaminating crop commodities before harvest, at harvesting and storage, as well as processed food products. Mycotoxins produce a wide range of adverse and toxic effects in animals in addition to being foodborne hazards to humans. The major fungal genera producing mycotoxins include *Aspergillus, Fusarium* and *Penicillium*, while mycotoxins of major concern worldwide are aflatoxins, ochratoxin A, fumonisins, deoxynivalenol, zearalenone, T-2 and HT-2 toxins [8,9]. To protect human health from exposure to these mycotoxins through the consumption of food products, the European Commission has established regulatory limits or recommendations for mycotoxins in raw materials and products intended for human and animal consumption [10,11,12,13]. Over the past decade, high-affinity and specific aptamers for a variety of mycotoxins have been reported, and in some cases, different aptamers for the same target mycotoxin have been reported in literature by different authors [4]. The first aptamer for mycotoxins was that reported by Neoventures Biotechnology Inc. (Canada) for ochratoxin A (OTA) [14]; next, three different research groups reported other ssDNA sequences candidates for OTA [15,16,17]. Furthermore, in addition to the first aptamer for aflatoxin B1 (AFB1) selected by the same company Neoventures Biotechnology [18], further aptamer sequences were described for AFB1 by other research groups [19,20,21,22,23,24]. Similarly, in addition to the first aptamer for fumonisin B1 (FB1) selected by McKeague et al. [25], new aptamer sequences were selected and described by other research groups [26,27,28]. The binding studies of these sequences showed binding affinity values, typically measured by the equilibrium dissociation constant (*k_D_*), in the nanomolar range. To date, these aptamers have been integrated into several applications for mycotoxin analysis, including colorimetric, electrochemical, electrochemiluminescence and fluorescent biosensors, enzyme-linked assays and affinity chromatography approaches. Extensive reviews have been recently published covering the recent developments of aptamer-based biosensors for the detection of mycotoxins [29,30,31,32].

Regardless of the intended application, high target affinity is a critical requirement of aptamers. However, aptamers with high affinities are not always isolated by conventional SELEX and several analytical methods have been proposed to assay and compare the different aptamer candidates. These technologies are costly and not always available in standard molecular biology laboratories where SELEX is typically performed [31]. Furthermore, these challenges are particularly problematic in the context of small molecule-binding aptamers, like mycotoxins, because most affinity binding assays are not sufficiently sensitive to measure the interaction of low molecular weight targets with the aptamer [33,34]. To overcome these limitations, several computational methods have been described in literature with the aim of streamlining the screening and selection of the aptamer during or post-SELEX [35,36]. Furthermore, these computational methods are becoming a method of choice, due to their convenience and low cost of investment in terms of chemical usage and logistics. To the best of our knowledge, the only applications of in-silico models to the screening of aptamers for mycotoxins was reported for the first time by Zhang et al. [37] who performed an in-silico docking study to investigate the binding mechanism between zearalenone and the relative aptamer. Afterwards, Mousivand et al. [38] improved affinity and selectivity of a known aptamer towards AFB1 after modifying it through a genetic algorithm based on in-silico maturation strategy.

The aim of the present paper is to describe an in-silico approach for rapid screening of three different families of ssDNA aptamer sequences against AFB1, FB1 and OTA. The agreement of the theoretical results with the measurements obtained for the same aptamers through binding experiments, using fluorescent microscale thermophoresis and a magnetic beads assay, allowed to demonstrate the applicability, reliability and usefulness of the proposed computational approach.

## 2. Materials and Methods

### 2.1. Reagents and Apparatus

The ssDNA sequences of aptamers FB1_39 [25], FB1_39t3 [26], FB1_10 [27], AF_AB3 [20] and AF_APT1 [19], and all aptamers modified with 5′-6-carboxyfluorescein were purchased from Bio-Fab Research Srl (Rome, Italy) as lyophilised form and with HPLC purity. Then, each aptamer was solubilized in the respective buffer solution. The ssDNA aptamer sequences for FB1 and AFB1 and the relevant composition of buffer solutions are reported in Table 1. All chemicals for buffer solutions and all other purposes were purchased from Sigma-Aldrich (Milan, Italy). The Dynabeads™ M-270 Amine and the DynaMag™-2 Magnet were obtained from Invitrogen (Life Technologies, Monza, Italy). FB1 and AFB1 standards were purchased from Vinci-Biochem Srl (Florence, Italy).

Mycotoxins stock solutions were prepared by dissolving AFB1 powder in toluene:acetonitrile (90:10, *v*/*v*) at concentration of 0.5 mg/mL, FB1 powder in acetonitrile:water (1;1, *v*/*v*) at concentration of 1 mg/mL and OTA powder in toluene:acetic acid (99:1; *v*/*v*) at concentration of 0.5 mg/mL. Mycotoxins working solutions were prepared by drying appropriate aliquots of stock standard solutions and redissolving them in the respective buffer solutions at concentrations ranging from 0.1 nM to 200 µM. Information relevant to OTA aptamers and relative standard preparation are reported elsewhere [31,39].

### 2.2. In-Silico Pipeline for Aptamer Screening

#### 2.2.1. Hardware and Software

For the aim of the present research, the following free computational software were chosen: SimRNA [40,41], RxDock [42], BioPython [43] and Open Babel [44]. SimRNA and RxDock where run on a 24 Intel^®^ Xeon^®^ E5-2640 core machine. All the computations were bounded by central processing unit (CPU) speed. The relevant timings can be found in the Supplemental Information (S.I.).

#### 2.2.2. Prediction of the 3-D DNA-Aptamer Structures

**Figure 1:**
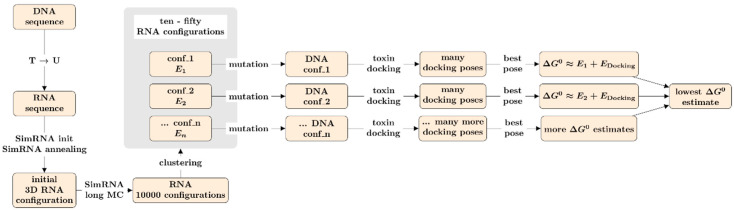
Scheme of the computational pipeline bringing from aptamer sequence to affinity estimate.

Our protocol for the determination of the three-dimensional structure of ssDNA aptamers consisted of six consecutive steps as shown in the procedural scheme of Figure 1. First, the DNA sequence of residues was mutated to RNA by changing thymine to uracil nucleotides. Second, the RNA structure was annealed by means of SimRNA. This software can build an initial regular, essentially cyclical, arrangement of RNA coil from the sequence, even though, this is very far from realistic, therefore a long annealing process lets this evolve towards the stability region of the potential landscape. Third, a large Monte Carlo dynamic was performed at normal temperature (T = 1 in SimRNA units). The simulation consisted of 50 M steps, with frames being saved every 5000 steps. Fourth, the resulting 10,000 three-dimensional structures were classified into a small number of clusters. On the coarse-grained representation of SimRNA, a clustering parameter of 7 Å brings to 2–50 clusters per molecule. For each cluster, only the molecule with lowest energy was considered for further treatment. Fifth, the coarse-grained representation was translated back to an all-atom representation. We used the program SimRNA_trafl2pdbs, which was found bundled with SimRNA. The translation can bring to some deviation of the P-O5’ bond between residues from the normal distance, sometimes as large as 0.5 Å, so that some software does not correctly recognize the chain as a single piece. Aside from problems with visualization software and interfaces to other computational programs, these deviations, that could be reduced with the help of molecular mechanics minimization, should not produce major effects on the length scale of docking potentials. Finally (sixth), the resulting RNA structures were mutated to DNA structures. A Python script, provided in the S.I., making use of the BioPython library [43], was written for the purpose. The program builds a standard Protein Data Bank (PDB) description of the residues, e.g., compatible with Gromacs expectations for the Assisted Model Building and Energy Refinement (AMBER) force fields.

#### 2.2.3. The Docking Approach

For each DNA molecule, the most stable configuration along with all similar configurations within 3 kcal/mol, were further processed and docked with the relevant toxin ligand molecule. To this purpose we used RxDock [42,45]. This software requires that the receptor and ligand be in specific formats, respectively, Sybil mol2 and MDL mol format. The conversion was achieved by means of Open Babel [44], which also adjusted the hydrogen atoms. RxDock defines the scanning room for docking as a sphere. However, defining a single sphere encompassing the entire receptor, tends to produce very large grids for ssDNA aptamers, which usually are not very compact. Indeed, the largest of our samples had for example an extension of almost 140 Å. To address this issue, a program was written that classifies the residue positions of a DNA configuration into a small number of clusters. For each of these a small sphere was obtained, typically of 15–25 Å, to which we added a 5 Å padding region.

RxDock allows customization of the docking through parameter files detailing the scoring function and each of the intermediate phases of the docking search, even providing some stock files for predefined purposes. For the present work, the complete docking protocol of RxDock was used, with the default parameters as detailed in dock.prm, coming with the RxDock package [42].

Docking trials were also carried out to test the selectivity of investigated aptamers towards mycotoxins different from their cognate target.

#### 2.2.4. Docking Calculations and Relation to Experimental Data

Docking calculations are partially driven by random number generation. Replicating the calculation several times provides information about the effectiveness of space exploration and, hence, the quality of the resulting poses. This is basically reflected in a distribution of docking scores.

Our strategy for deciding the number of repetitions consisted in obtaining at least four replicas yielding docking scores within 3 kcal/mol of the lowest value. This means that in the worst and unrealistic case of a uniform docking score distribution, the probability to find a pose better than those already obtained by more than 3 kcal/mol was less than 1/8. However, for a more realistic bell-shaped distribution, such probability is actually much lower.

In the case of OTA and AFB1 docking, the distribution was extremely tight and most repetitions yielded identical optimal results, therefore, the number of repetitions was fixed to 5. For FB1 the docking score distribution was extremely wide, therefore, to meet the mentioned criterion about 100 repetitions were needed.

The best docking score was then added to the aptamer configurational energy, yielding what we for simplicity call a ΔG^0^ estimation (though actually more related to enthalpy), that was correlated with the experimental binding ΔG^0^. This is of course straightforwardly obtained from dissociation constant (*K_D_*) values through the relation ${∆G}^{0}=-RTln\left(K_{D}\right)$.

### 2.3. Experimental Assays

#### 2.3.1. Fluorescent Microscale Thermophoresis (MST)

Binding assays by fluorescent Microscale Thermophoresis (MST) for FB1 and AFB1 aptamers were carried out by the company 2Bind, GmbH (Regensburg, Germany), according to the procedure described by McKeague et al. [39]. Briefly, 5 µL of each serial dilution of standard mycotoxin (from 1.464 nM to 48 µM) in the appropriate binding buffer were mixed with 5 µL of 10 nM of fluorescently labelled aptamer. The final reaction mixture, containing a constant concentration of fluorescent aptamer (5 nM) and a variable mycotoxin standard solution (ranging from 0.732 nM to 24 µM) was filled in capillaries. Samples were analyzed on a Monolith NT at 25 °C, with 35% LED power and 80% Laser power. The binding between aptamer and mycotoxin was monitored by measuring the changes in the thermophoretic behavior through a temperature gradient. For each aptamer two independent experiments were carried out at each tested concentration. The dissociation constant *K_D_* value (highest affinity, lowest *K_D_*) was obtained by fitting the binding curve with the fraction of fluorescent aptamer that formed the complex, calculated for each independent experiment (*n* = 2) from the law of mass action [46]. Details on the MST assay for OTA aptamers are reported in a paper by McKeague et al. [39].

#### 2.3.2. Magnetic Beads (MBs) Assay

The functionalization of magnetic beads with FB1 was performed according to the procedure described by Frost et al. [26]. Briefly, an aliquot (450 µL) of amine-activated Dynabeads^®^ (10^8^ beads, 30 mg/mL) was washed three times with PBS buffer (50 µL) and separated on the magnet each time. Then, the beads were re-suspended with 5% glutaraldehyde (Sigma Aldrich) in PBS buffer and incubated (for 2 h at room temperature) under slow stirring. After three washes with PBS buffer, the beads were re-suspended into 450 µL of FB1 standard (20 μM) in PBS buffer solution, and incubated (at room temperature) under slow stirring overnight. At the end of the process, unbound NH_2_ groups, still present on the bead surface, were capped by incubation with a solution of Sulfo-NHS acetate (Thermo Scientific) in buffer (for 2 h at room temperature) under slow stirring. The beads were washed other three times with PBS buffer and stored at 4 °C till use. All the washing solutions from the coupling were collected and analyzed by HPLC for unbound FB1 quantification according to De Girolamo et al. [47]. The analyses indicated that more than 99% of FB1 was conjugated to the beads.

The functionalization of magnetic beads with AFB1 was performed according to the manufacturer’s protocol (Invitrogen by Life Technologies) and the coupling was achieved by Schiff base (imine) formation and reductive amination. Briefly, an aliquot (300 µL) of amine-activated Dynabeads^®^ (10^8^ beads) were washed three times with 0.1 M sodium borate buffer, pH 9.4 (300 µL) and separated on the magnet each time. The washed beads were mixed with 300 µL of AFB1 standard solution (180 µM) in 0.1 M sodium borate buffer; then, an aliquot (6 µL) of 5 M sodium cyanoborohydride in 1 M NaOH was added to the reaction mixture, vortexed and incubated (for 2 h at room temperature) under slow stirring. After removal of supernatant, an aliquot (600 µL) of 0.1 M ethanolamine (pH 7.4) was added to the reaction mixture and incubated (for 15 min at room temperature) by gentle mixing. The coated beads were then washed three times with 0.5% BSA, 0.01% Tween^®^-20, and 0.02% sodium azide, and beads were stored in buffer (100 mM NaCl, 20 mM Tris–HCl (pH 7.6), 2 mM MgCl_2_, 5 mM KCl, 1 mM CaCl_2_ at 4 °C) at 4 °C until use. All the washing solutions collected after the functionalization reactions were collected and analyzed by fluorescence spectroscopy (LS-55, Perkin Elmer, Waltham, MA, USA) for AFB1 quantification using a standard 10 mm path length quartz cuvette. Spectra were acquired in the 350–600 nm spectral range with λ_ex_ set at 494 nm and λ_em_ set at 520 nm. It was calculated that more than 99% of the mycotoxin was conjugated to the beads.

The affinity of the selected aptamers for their target mycotoxin was determined by incubating increasing concentrations of 5 ’FAM labelled aptamer (100 µL) prepared in the respective buffer with a fixed amount of mycotoxin-coupled beads (25 µL). In particular, concentrations were between 0.5–100 nM for aptamer FB1_39, between 5–2000 nM for aptamer FB139_t3, between 20–2000 nM for aptamer FB1_10 and between 0.5–250 nM for AF_Apt1, between 0.5–4000 nM for aptamer AF_AB3. After a 1-h binding reaction (at room temperature) the unbound aptamers were removed by several washing steps with binding buffer. The amount of DNA aptamer eluted from the beads was determined by fluorescence measurements with λ_ex_ set at 494 nm and λ_em_ set at 520 nm and calculations using a calibration plot. For each aptamer two independent experiments were carried out. The value of K_D_ was calculated by applying a nonlinear regression equation using the function of “receptor for a non-specific binding site” (SigmaPlot v. 12.3). Details on the MBs assay for OTA aptamers are reported in the paper by McKeague et al. [31].

## 3. Results and Discussion

In the present study three families of aptamers for three target mycotoxins, namely, fumonisin B1 (FB1), aflatoxin B1 (AFB1) and ochratoxin A (OTA), were screened by using an in-silico pipeline approach for their binding affinity towards target mycotoxins. The reliability of the proposed approach was verified by comparing theoretical results with those obtained by using two experimental approaches, i.e., Fluorescent Microscale Thermophoresis (MST) and Magnetic Beads (MBs) assays.

### 3.1. In-Silico Binding Affinity of Mycotoxins to Aptamers

The strategy presented herein for gauging the binding affinity of an aptamer–toxin couple consists of obtaining a set of possible conformations for the aptamer, within a reasonable range of conformational energies. Then, the toxin is docked to the aptamer to obtain a docking score that summed to the conformational energy provided an estimate of the binding free energy.

As much as the first task is concerned, i.e., obtaining the three-dimensional aptamer configurations, our six-step procedure may look somewhat convoluted. The problem is that, to our knowledge, there is no software that straightforwardly addresses this problem for ssDNA. Contrarily, there is a good choice of software specialized in making previsions of structure for RNA fragments. Therefore, we were somewhat obliged to take an indirect route, that is, studying a corresponding RNA fragment and then rigidly mutating this to a DNA fragment by leaving fixed all the atoms in common. Zhang et al. [37] opted for a similar strategy, but used Mfold and RNA Composer for the realization of RNA tertiary structure. Our choice, SimRNA, though certainly more computationally expensive, presents a distinct advantage.

The main duty of SimRNA is to perform a Monte Carlo dynamic of a simplified, coarse grained representation of an RNA fragment. Such coarse-grained representation consists of 5–6 freedom degrees per residue, dealing with nucleotides as essentially rigid entities. The energy landscape is provided by a model potential, the most important terms of which were written so that simulations reproduce geometrical relationships observed in nature. These were extracted from a curated database of experimental well-resolved structures. The main effect of this approach is that SimRNA provides an evolving view of the molecule rather than a single geometry. The molecule movie is then characterized with clustering techniques, by means of which characteristic configurations are extracted. While all this results in extra work, it may produce more reliable results, considering the fact that the subsequent docking is conducted holding the aptamer rigid. For example, for aptamer FB1_39, we considered four different configurations. According to SimRNA, these configurations are pretty much equally plausible, can be found with similar frequency during the simulation and, possibly, are all similarly present in a real solution.

As for most docking software, the RxDock scoring function is a sum of interaction energies between receptor and ligand. These are expressed in kcal/mol and are meant to mimic the enthalpy of the binding process: the lower the value, the better the binding. It must be noted that, notwithstanding the physics-based recipe behind it, the primary purpose of the scoring function for RxDock is to provide a ranking of the docking poses and not to provide an energetic estimate of the binding process. In the same way, the main role of the SimRNA potential for SimRNA is to assess a relative probability of a configuration compared to another, the authors of SimRNA are explicit about this. Therefore, while our estimate of the binding affinity as E_dock_ + E_SimRNA_ is somewhat natural and obligatory, its correspondence to ΔG^0^ cannot be taken for granted but needs to be verified by means of a sort of experimental verification.

**Figure 2:**
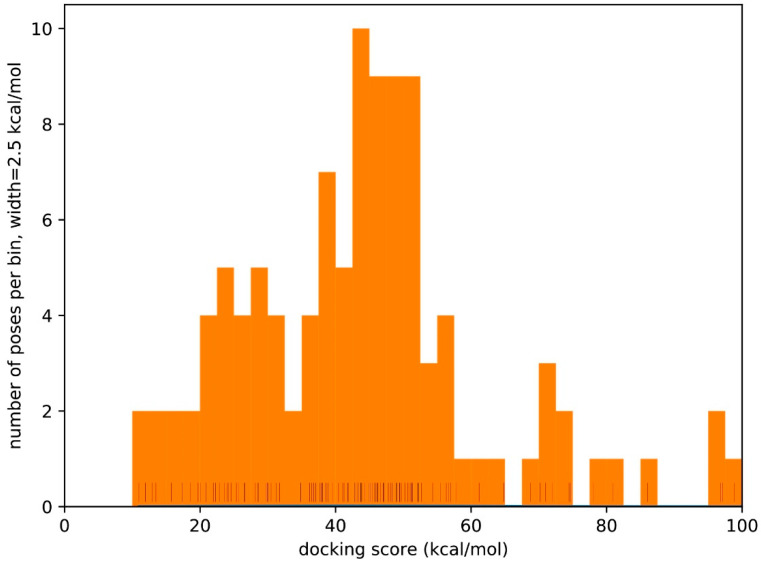
Histogram and rug plot of more than 100 different docking score results for the FB1_10-FB1 system.

Table 2 reports a set of predictions for each of the selected aptamers towards target mycotoxins, the relevant docking score values and the DNA configurational energy. In general, the pool of DNA configurations was restrained to those within 3 kcal/mol of the most stable one, but often only one configuration survives this selection criterion. However, in the case of FB1_10, we decided to include more configurations. For OTA and FB1, the docking energy reported pertains to the best of five different docking runs. This repetition was deemed necessary because, depending on the exhaustiveness of the configuration space sampling, docking calculations are not necessarily deterministic; indeed, docking calculations somewhat rely on random number generation. However, in the case of OTA and FB1, the resulting docking calculation was highly reproducible, with small dispersion and rare cases of badly guessed poses. Figure 2 shows a histogram of the docking score values for more than one hundred calculations of the FB1_10-FB1 system in a docking sphere of 15.1 Å, with identical input. The dispersion was very high, so that only five poses were within the lowest 5 kcal/mol. Therefore, one can expect so many samples to be necessary to obtain reliable results for systems like fumonisins. This is probably due to the large number of free dihedrals and therefore configurational space of such molecules. Incidentally, each run took 2–3 times more CPU time for these flexible molecules.

**Figure 3:**
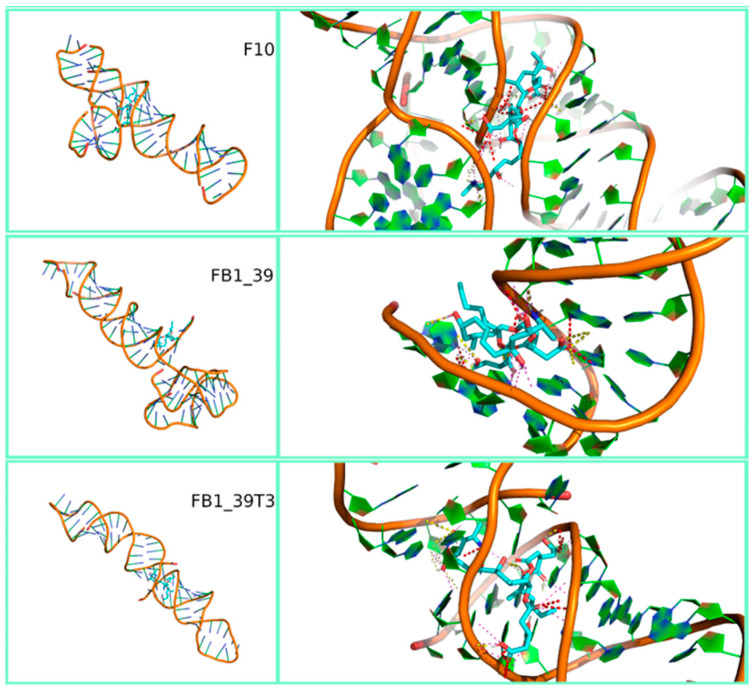
Three-dimensional representation of the best-docked pose of the three fumonisin B1 aptamers (F_10, FB1_39 and FB1_39t3). Full view on the left and zoom with contact maps on the right.

**Figure 4:**
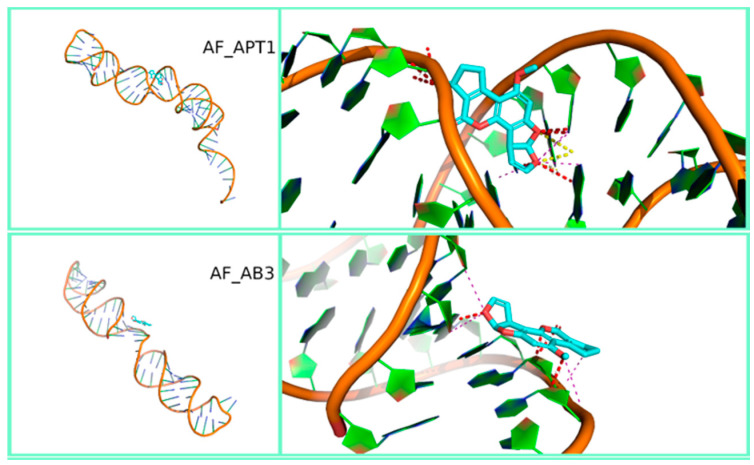
Three-dimensional representation of the best-docked pose of the two aflatoxins B1 aptamers (AF_APT1 and AF_AB3). Full view on the left and zoom with contact maps on the right.

**Figure 5:**
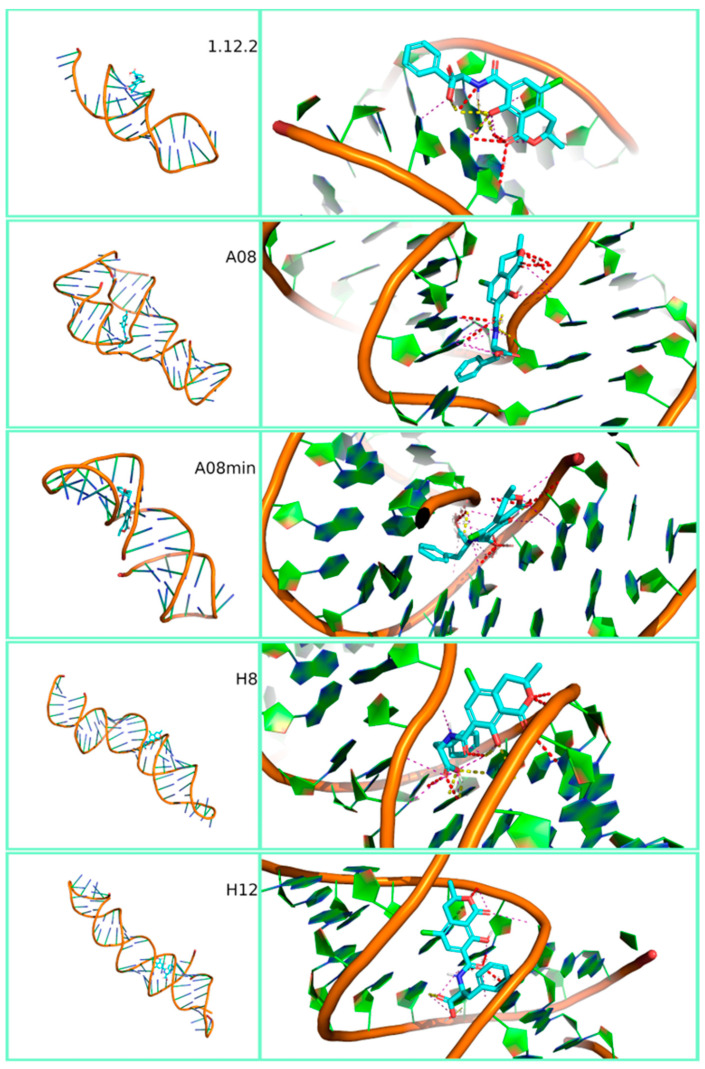
Three-dimensional representation of the best-docked pose of the five ochratoxin A aptamers (1.12.2, A08, A08min, H8 and H12). Full view on the left and zoom with contact maps on the right.

Figure 3, Figure 4 and Figure 5 show the most stable docked poses for FB1, AFB1 and OTA aptamers, respectively. A large variety of binding possibilities were obtained, with hydrogen bonding, hydrophobic repulsion and other kinds of electrostatic interactions being involved with differing number and intensity and no road to a simple or uniform interpretation of the interaction energy. Fumonisins, however, evidenced a higher predisposition for bonding to the aptamer backbone. Notably, the majority of these aptamer structures compare well with what has been predicted for the sequences. For example, circular dichroism experiments on FB1_39 show indications of a B form duplex structure [48], which is partially supported in our modelling (Figure 3). Note that RNA prefers to adopt an A-form duplex which would account for the differences in our model. In contrast, in the case of OTA aptamers, while the modelling of aptamer 1.12.2 predicts a stem-loop with a short single-stranded overhang (Figure 5), circular dichroism experiments show the characteristics of an anti-parallel G-quadruplex structure [49]. This discrepancy could be due to the difficulty in modelling G-quadruplex structures by SimRNA and should be kept under consideration when comparing binding affinity results.

Aptamer selectivity is also an important characteristic that needs to be understood before a sequence can be determined to be fit for purpose. The queried aptamer systems should all be expected to show limited cross-reactivity to mycotoxins other than their cognate target. The docking of AFB1 with all the OTA-binding aptamers revealed docking energies that were consistently less stable for AFB1, as expected (Table S2). In particular, A08 and A08min showed a destabilization of 4.28 and 6.95 kcal/mol, respectively, when the incorrect target was docked. Considering that a difference of 1 kcal/mol equates to a factor 5.4 in the binding constant, the selectivity of the OTA-binding aptamers is confirmed. In contrast, when OTA was docked onto the AFB1-binding sequences, similar or improved docking scores were observed (Table S1). This finding is in agreement with previous studies reporting a comparable or slightly lower selectivity towards other target mycotoxins for aptamers specific for AFB1 [18,37]. Further computational investigations will be carried out to perform other cross-reactivity studies to predict the affinity of the investigated aptamers in situations which may arise in multi-mycotoxin-contaminated samples. Further investigations will focus also on verifying if the AF_AB3 and AF_APT1 aptamers use a different binding site for OTA compared to that of AFB1.

### 3.2. Experimental Binding Affinity of Aptamers to Mycotoxins

Fluorescent Microscale Thermophoresis (MST) is a recently described method that leverages the physical phenomenon of molecular movement within temperature gradients. Each molecule (or aptamer) has a very specific “thermophoresis” that is dependent on the size, charge and hydration shell; therefore, upon binding to a target molecule, at least one of these parameters will be altered and can be measured. Because the movement of the interacting partners can be monitored with fluorescence, in-solution binding information can be obtained [50]. MST assay was chosen as an experimental approach because it is a powerful tool to characterize aptamer–target interactions independent in a highly sensitive cost- and time-efficient performance and with a free choice of buffers. Furthermore, the low sample consumption and the flexible assay setup are great advantages of this in-solution method [50]. Binding assays results by MST indicated a good binding of aptamers FB1_39 and FB1_10 towards FB1 and of aptamer AF_AB3 towards AFB1, with *K_D_* values lower than 200 nM, even though a certain variability of results was observed (up to 85%). This variability was probably related to the poor solubility of the mycotoxins in the buffer solutions at the tested concentrations. The aptamer AF_APT1 did not show any binding towards AFB1 (Table 3). In the case of OTA aptamers, all of them showed a good binding affinity towards the target mycotoxin in the nanomolar range, with A08min showing the highest affinity as previously reported [39] (Table 3).

Magnetic beads (MBs) assay is a fast and easy-to-use tool for isolation of analytes from complex matrices thanks to the colloidal stability of magnetic nanoparticles, homogenous size distribution, high and uniform magnetite content and presence of surface functional groups [30]. Results of the MBs assay confirmed the good binding affinity of aptamer FB1_39 towards FB1, while the other two aptamers, i.e., FB1_39t3 and FB1_10, did not show any binding towards FB1. Conversely to results obtained from MST assay, a good binding affinity towards AFB1 was obtained for AF_APT1, while aptamer AF_AB3 did not show any binding towards AFB1. All the aptamers towards OTA showed *K_D_* values comparable to those obtained by MST [31,39] (Table 3).

The different results observed herein between the aptamers belonging to the same family could be explained by the typology of the assays. Indeed, a previous study carried out by McKeague et al. [44] demonstrated that a given aptamer may have limitations or opportunities that make it better suited for certain molecular recognition schemes or applications. In the present study, in the MST assay both the target mycotoxin and the aptamer were free in solution, while the MBs assay was based on the interaction between free aptamer in-solution and bounded mycotoxin on the beads’ surface. The fact that the binding studies carried out by MBs did not show any binding for three out five aptamers as compared to MST assay indicated that the labelling at 5′ end of the aptamer may have abolished the binding ability of the aptamer (Table 3). Furthermore, when the target mycotoxin is bounded on a surface, the assays may suffer from nonspecific bindings between the aptamers and the surface, thus reducing their functionality in the investigated application. These results were in agreement with those reported by McKeague et al. [31,44] that performed a comparison of aptamer binding assays for a family of seven different aptamers towards the OTA using eight different assays, including MBs. As important differences beyond binding affinity between the three families of aptamers were observed, they concluded that aptamer binding varied when either the target or aptamer is used in solution vs. immobilized, and the sensitivity of each technique affected the apparent affinity [31,39]. A similar variability in terms of *K_D_* values calculated by using three different experimental methods was also reported by Mousivand et al. [38] for a family of six aptamers against AFB1. Altogether these results indicate that to screen aptamers it is important to implement high-throughput methods in order to screen more candidate ones.

In general, methods allowing aptamer–target binding occurring free in solution, as the MST assay described herein, are the preferred ones because they remove any contribution from matrix binding responsible for reducing aptamer functionality. On the other hand, colorimetric assays, like the MBs one, are widely applied in quantitative detection of mycotoxins, especially in the real-sample applications [29,30,32].

### 3.3. Comparison of In-Silico and Experimental Binding Affinity of Aptamers to Mycotoxins

Characterization of aptamer binding is critical for studying aptamer molecular recognition and integrating them into diverse applications. In the current decade, the screening of aptamers through computational docking and molecular dynamics study has greatly increased and the application for computational prediction of the aptamer affinity against its specific target is becoming a commonly used procedure [35]. Indeed, the limitation in terms of costs of the high-throughput screen of aptamers through experimental trials are overcome by in-silico methods that allow to rapidly and reliably screen aptamer candidates.

To compare the in-silico prediction results with the experimental ones for each aptamer–toxin couple, the *K_D_* results, calculated by using MST and MBs assays and expressed as nmol/L values, were translated to free energy ones by means of the relation ${∆G}^{0}=-RTln\left(K_{D}\right)$ formula, where T is held at 298° K. This, as discussed above, is then compared to an estimate of ΔG^0^ obtained as E_dock_ + E_conf_.

**Figure 6:**
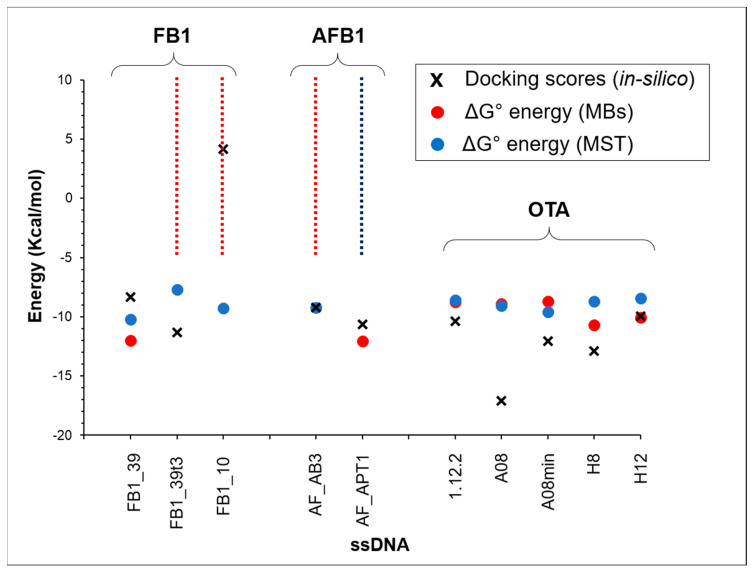
Comparison of experimental affinity values (ΔG^0^ energy, kcal/mol) and in-silico affinity prediction (best estimated ΔG^0^, kcal/mol) as obtained from docking score plus configurational aptamer energy. The dashed line indicates the lacking data for MST (blue line) or MBs (red line) assay.

Figure 6 is a visual representation of the comparison, overlapping for each aptamer the experimental results from MST and MBs tests and the in-silico prediction. Vertical dashed lines were used to describe those cases where *K_D_* and the corresponding binding energy are above the technique measurement limit. The agreement between simulation and experimental data is almost quantitative, however, predictions for FB1, and in particular its binding to FB1_10 deserve a few considerations. In general, such model calculations as those in the present paper, mostly presuppose that the system is free in solution. This is not granted under all respects. For example, as said above, the SimRNA potential is derived from experimental structural data, most of which certainly do not refer to dilute RNA solutions. In most cases, the experimental MST and MBs affinities are similar, making the comparison more straightforward. However, as explained above, for AF_AB3, FB1_10 and FB1_39T3, MBs is not able to detect any binding. Moreover, in the case of AF_APT1, MST gives a negative response. In these limited four out of ten cases, we cannot make any quantitative comparison. However, the calculated prediction for FB1_10 is certainly suspect. In the case of FB1_10 we also relieved the sampling constraints on conformations to extend the study to more improbable ones, but could not detect any effective binding. More in general, a more accurate benchmark is needed for molecules like fumonisins, both to assess the reliability of calculations and to improve the efficiency of the simulation. Basically, when the MST and MB assays give similar or strictly correlated affinity values, as is the case for OTA, one can avoid pondering whether the in-silico data are better compared to the first or the latter set of experimental data, a question which is not easily addressed because of the sources of the SimRNA potential. On the other hand, AFB1 comes with two aptamer samples, one with MB good affinity and very poor MST affinity, the other doing the exact opposite.

A possible explanation of the more homogeneous behavior of the five OTA aptamers with the two different experimental approaches could be related to their secondary structure. Indeed, the sequence analysis of OTA aptamers by using the “m-Fold” program revealed in all the five aptamers two conserved consensus sequences. The first sequence (GGGTGTGGG) was located in the steam region, which stabilizes the whole structure due to high GC contents, while the second one (AGGGAGT) was located in the in the single-strand terminal loop. Both these two regions were most likely responsible for the binding with OTA independently from the surrounding in which the recognition takes place [16]. In the case of AFB1 and FB1 aptamers investigated herein, none of these or other conservative sequences were observed in their structure, as well as, a lower content of GC was present. Both these factors could be responsible of the minor stability of these families of aptamers in the tested conditions and then, to the greater lack of homogeneity of the results. A further confirmation of these results comes from the numbers of applications reported in literature for these three families of aptamers. In particular, the most numerous applications to food samples contaminated with mycotoxins were described for OTA aptamers followed by AFB1 aptamers and to a lesser extent for FB1 aptamers [29,30,32], thus suggesting the major versatility of OTA and AFB1 aptamers compared to the other ones.

## 4. Conclusions

In the present study we propose for the first time an in-silico pipeline approach for the rapid screening of different ssDNA aptamers against three mycotoxins such as fumonisin B1, aflatoxin B1 and ochratoxin A. Furthermore, the comparison of the outputs of the theoretical screening with experimental ones was a useful strategy to validate the theoretical results. The proposed in-silico method for affinity prediction is conveniently cheap, requiring modest computational resources and being easily automatable to cope with large aptamer databases. With the current parameters, in general, good agreement of theoretical results with those obtained with experimental assays was observed and in particular with MST assay in which both aptamer and target mycotoxin are free in solution. Furthermore, it worked far more reliably and efficiently for aflatoxin B1 and ochratoxin A and to a lesser extent with fumonisin B1, and therefore, plausibly, with similar molecules, endowed with a large number of free dihedral angles. It is, however, reasonable that a strict adaptation of the RxDock docking protocol could provide good results also for this class of molecules.

The consistency of the in-silico results with the experimental findings confirmed that this strategy is a promising tool to be used for the screening of aptamer affinity towards small molecules like mycotoxins, even though the selectivity of aptamers observed by computational methods should be confirmed by experimental tools.

## Figures and Tables

**Table 1:** Fumonisin B1 (FB1), aflatoxin B1 (AFB1) and ochratoxin A (OTA) aptamers and relative buffer composition.

Target Mycotoxin	Aptamer Name	ssDNA Sequence (5′-3′)	Buffer Composition	Reference
**FB1**	FB1_39	ATACCAGCTTATTCAATTAATCGCATTACCTTATACCAGCTTATTCAATTACGTCTGCACATACCAGCTTATTCAATTAGATAGTAAGTGCAATCT	100 mM NaCl, 20 mM Tris, 2 mM MgCl_2_, 5 mM KCl, 1 mM CaCl_2_, pH 7.6	[25]
	FB1_39t3	ATACCAGCTTATTCAATTAATCGCATTACCTTATACCAGCTTATTCAATTACGTCTGCACATACCAGCTTATTCAATT	Same as FB1_39	[26]
	FB1_10	AGCAGCACAGAGGTCAGATGCGATCTGGATATTATTTTTGATACCCCTTTGGGGAGACATCCTATGCGTGCTACCGTGAA	100 mM NaCl, 20 mM Tris–HCl, 2 mM MgCl_2_, 5 mM KCl_,_ 1 mM CaCl_2_, 0.02 % Tween 20 (pH 7.4)	[27]
**AFB1**	AF_AB3	ATCCGTCACACCTGCTCTATTCCTCTGTTGAAGAACCACTTCCGGAAATAAGAGTGGTGTTGGCTCCCGTAT	100 mM NaCl, 20 mM Tris–HCl (pH 7.6), 2 mM MgCl_2_, 5 mM KCl, 1 mM CaCl_2_	[20]
	AF_APT1	AGCAGCACAGAGGTCAGATGGTGCTATCATGCGCTCAATGGGAGACTTTAGCTGCCCCCACCTATGCGTGCTACCGTGAA	100 mM NaCl, 20 mM Tris–HCl (pH 7.6), 2 mM MgCl_2_, 5 mM KCl, 1 mM CaCl_2_, 0.02% Tween 20 (pH 7)	[19]
**OTA**	1.12.2	GATCGGGTGTGGGTGGCGTAAAGGGAGCATCGGACA	10 mM HEPES, 120 mM NaCl, 5 mM KCl, 10mM CaCl_2_, pH 7.0	[14]
	A08	AGCCTCGTCTGTTCTCCCGGCAGTGTGGGCGAATCTATGCGTACCGTTCGATATCGTGGGGAAGACAAGCAGACGT	10 mM Na_2_HPO_4_, 2 mM KH_2_PO_4_, 137 mM NaCl, 2.7 mM KCl, pH 7.4	[15]
	A08min	GGCAGTGTGGGCGAATCTAT GCGTACCGTTCGATATCGTG	Same as A08	[15]
	H8	GGGAGGACGAAGCGGAACTGGGTGTGGGGTGATCAAGGGAGTAGACTACAGAAGACACGCCCGACA	10 mM Na_2_HPO_4_, 2 mM KH_2_PO_4_, 137 mM NaCl, 2.7 mM KCl, 1 mM MgCl_2_, pH 7.4	[16]
	H12	GGGAGGACGAAGCGGAACCGGGTGTGGGTGCCTTGATCCAGGGAGTCTCAGAAGACACGCCCGACA	Same as H8	[16]

**Table 2:** Docking score values and relative DNA configurational energy calculated by using the in-silico approach for each of the selected aptamer towards target mycotoxin. ΔG^0^ is estimated as the sum of docking score and configurational energy.

Target Mycotoxin	Aptamer Name	Docking Score (kcal/mol)	Configurational Energy (kcal/mol)	Best Estimated ΔG^0^ (kcal/mol)
Fumonisin B1 (FB1)	FB1_39	−5.50 6.70 −9.04 −9.25	0.0 0.1 0.7 1.6	−8.34
	FB1_39t3	−11.33	0.0	−11.33
	FB1_10	10.98 1.19 −5.58	0.0 4.89 9.76	4.18
Aflatoxin B1 (AFB1)	AF_AB3	−9.20	0.0	−9.20
	AF_APT1	−10.66 −11.16 −11.72	0.0 0.8 1.9	−10.66
Ochratoxin A (OTA)	1.12.2	−10.38	0.0	−10.38
	A08	−17.08	0.0	−17.08
	A08min	−12.05	0.0	−12.05
	H8	−12.91	0.0	−12.91
	H12	−9.98	0.0	−9.98

**Table 3:** Dissociation constant (*K_D_*) values and corresponding free energy (ΔG^0^) values calculated by using Fluorescent Microscale Thermophoresis (MST) and magnetic beads (MBs) assays of selected aptamer towards target mycotoxins. The standard error values of the calculated *K_D_* values were derived from two independent experiments.

Target Mycotoxin	Aptamer Name	MST Assay		MBs Assay	
		*K_D_* (nmol/L)	ΔG^0^ Energy ^a^ (kcal/mol)	*K_D_* (nmol/L)	ΔG^0^ Energy (kcal/mol)
**Fumonisin B1** **(FB1)**	FB1_39	31 ± 22	−10.24 ± 0.42	1.53 ± 0.67	−12.03 ± 0.26
	FB1_39t3	2200 ± 1100	−7.72 ± 0.30	NB ^b^	- ^c^
	FB1_10	162 ± 137	−9.26 ± 0.50	NB	-
**Aflatoxin B1** **(AFB1)**	AF_AB3	178 ± 50	−9.21 ± 0.17	NB	-
	AF_APT1	NB	-	1.40 ± 0.51	−12.08 ± 0.22
**Ochratoxin A (OTA)**	1.12.2	525 ± 147 ^d^	−8.57 ± 0.17	374 ± 255 ^e^	−8.77 ± 0.40
	A08	233 ± 81 ^d^	−9.05 ± 0.21	286 ± 149 ^e^	−8.93 ± 0.31
	A08min	97 ± 33 ^d^	−9.57 ± 0.20	406 ± 166 ^e^	−8.72 ± 0.24
	H8	416 ± 119 ^d^	−8.70 ± 0.17	14 ± 7 ^e^	−10.71 ± 0.30
	H12	630 ± 80 ^d^	−8.46 ± 0.08	40 ± 14 ^e^	−10.09 ± 0.21

^a^ Free energy values calculated according to the formula:${\;∆G}^{0}=-RTln\left(K_{D}\right);$ ^b^ NB = no binding detected; ^c^ not applicable; ^d^ McKeague et al. [39]; ^e^ McKeague et al. [31].

## Supplementary Materials

The following are available online at https://www.mdpi.com/2073-4360/12/12/2983/s1, Table S1: *In-silico* selectivity tests for aflatoxin B1 (AFB1) aptamers towards ochratoxin A (OTA) and comparison with docking calculations for the target AFB1. Table S2: *In-silico* selectivity tests for ochratoxin A (OTA) aptamers towards aflatoxin B1 (AFB1) and comparison with docking calculations for the target OTA. Table S3: Timings (s).
