# Supplementary material for: An In-Silico Pipeline for Rapid Screening of DNA Aptamers against Mycotoxins: The Case-Study of Fumonisin B1, Aflatoxin B1 and Ochratoxin A
Source: Polymers (Basel). 2020 Dec 14;12(12):2983. doi: 10.3390/polym12122983 (PMC7764985; doi:10.3390/polym12122983)
Supplement: Supplementary file 1 [file polymers-12-02983-s001.pdf]

An *in-silico* pipeline for rapid screening of DNA aptamers against mycotoxins: the case-study of fumonisin B1, aflatoxin B1 and ochratoxin A

Fulvio Ciriaco*[1], Vincenzo De Leo[1], Lucia Catucci[1], Michelangelo Pascale[2], Antonio F. Logrieco[2], Maria C. DeRosa[3], Annalisa De Girolamo*[2]

[1] Department of Chemistry, University of Bari, Via Orabona 4, Bari, I-70126, Italy; vincenzo.deleo@uniba.it (V.D.L.); lucia.catucci@uniba.it (L.C.).

[2] Institute of Sciences of Food Production (ISPA), CNR-National Research Council of Italy, Via G. Amendola 122/O, 70126 Bari, Italy; michelangelo.pascale@ispa.cnr.it (M.P.); antonio.logrieco@ispa.cnr.it (A.F.L.).

[3] Department of Chemistry, Carleton University, 1125 Colonel by Drive, Ottawa, ON, K1S 5B6, Canada maria_derosa@carleton.ca (M.C.D.).

* Correspondence: fulvio.ciriaco@uniba.it; Tel.: +39.080.544.2041 (F.C.)
annalisa.degirolamo@ispa.cnr.it; Tel.: +39.080.592.9351 (A.D.)

Programs, configuration files and actual input and outputs can be found at:
https://github.com/oivulf/dna_toxin_binding

Table S1. ***In-silico*** **selectivity tests for aflatoxin B1 (AFB1) aptamers towards ochratoxin A (OTA) and comparison with docking calculations for the target AFB1.**

	Docking energy (kcal/mol)	
**Aptamer name**	**AFB1**	**OTA**
AF_AB3	-9.2	-9.1
AF_APT1	-10.66	-12.6

Table S2. ***In-silico*** **selectivity tests for ochratoxin A (OTA) aptamers towards aflatoxin B1 (AFB1) and comparison with docking calculations for the target OTA.**

	Docking energy (kcal/mol)	
**Aptamer name**	**OTA**	**AFB1**
1.12.2	-10.38	-8.2
A08	-17.08	-12.8
A08min	-12.05	-5.1
H8	-12.91	-10.5
H12	-9.98	-8.9

Table S3. Timings (s)

Aptamer name	Annealing	Dynamics	Docking: grid generation (20 core)	Docking: space sampling, single pose, mean
**Fumonisin B1**				
FB1_39	41358	109729	720	6325
FB1_39T3	39000	78500	835	5131
FB1_10	49698	81250	735	1500
**Aflatoxin B1**				
AF_AB3	43272	69537	360	93
AF_APT1	49345	78681	280	105
**Ochratoxin A**				
1.12.2	17567	27912	481	123
A08	45211	71216	475	74
A08min	20900	34216	417	55
H8	38385	61370	312	55
H12	37150	60121	312	53
